# Methamphetamine Use Associated with Non-adherence to Antiretroviral Treatment in Men Who Have Sex with Men

**DOI:** 10.1038/s41598-020-64069-2

**Published:** 2020-04-28

**Authors:** Hsin-Hao Lai, Yen-Chun Kuo, Chian-Jue Kuo, Yun-Ju Lai, Marcelo Chen, Yi-Tui Chen, Chu-Chieh Chen, Muh-Yong Yen, Bor-Shen Hu, Teng-Ho Wang, Chien Chun Wang, Li-Lan Kuo, Tsen-Fang Yen, Pei-Hung Chuang, Yung-Feng Yen

**Affiliations:** 1 Section of Infectious Diseases, Taipei City Hospital, Yangming Branch, Taipei, Taiwan; 2 School of Medicine, National Yang-Ming University, Taipei, Taiwan; 3 Department of Psychiatry, Taipei City Hospital, Linsen, Chinese Medicine, and Kunming Branch, Taipei, Taiwan; 4 Taipei City Psychiatric Centre, Taipei City Hospital, Taipei, Taiwan; 5 Department of Psychiatry, School of Medicine, College of Medicine, Taipei Medical University, Taipei, Taiwan; 6 School of Medicine, National Yang-Ming University, Taipei, Taiwan; 7 Division of Endocrinology and Metabolism, Department of Internal Medicine, Puli Branch of Taichung Veterans General Hospital, Nantou, Taiwan; 8 Department of Exercise Health Science, National Taiwan University of Sport, Taichung, Taiwan; 9 Department of Urology, Mackay Memorial Hospital, Taipei, Taiwan; 10 Department of Cosmetic Applications and Management, Mackay Junior College of Medicine, Nursing and Management, Taipei, Taiwan; 11 Department of Health Care Management, National Taipei University of Nursing and Health Sciences, Taipei, Taiwan; 12 Section of Infectious Diseases, Taipei City Hospital, Taipei, Taiwan; 13 Section of Infectious Diseases, Taipei City Hospital, Heping Fuyou Branch, Taipei, Taiwan; 14 Section of Infectious Diseases, Taipei City Hospital, Zhongxiao Branch, Taipei, Taiwan; 15 Section of Infectious Diseases, Taipei City Hospital, Linsen, Chinese Medicine, and Kunming Branch, Taipei, Taiwan; 16 Department of Nursing, Taipei City Hospital, Linsen, Chinese Medicine, and Kunming Branch, Taipei, Taiwan; 17 Taipei Association of Health and Welfare Data Science, Taipei, Taiwan; 18 Institute of Public Health, National Yang-Ming University, Taipei, Taiwan

**Keywords:** Health care, Risk factors

## Abstract

Methamphetamine is a prevalent recreational drug among men who have sex with men (MSM) living with HIV and could cause the cognitive impairment and memory loss. However, studies on the association between methamphetamine use and adherence to antiretroviral treatment (ART) are limited and had inconsistent findings. This study aimed to determine the impact of methamphetamine use on adherence to ART among MSM living with HIV. From December 2018 to October 2019, MSM living with HIV were recruited (*N* = 351) and non-adherence to ART was defined as a Medication Adherence Report Scale score of <23. Overall, 16.0% of the participants reported methamphetamine use in the prior three months and 13.4% of the participants had non-adherence to ART. The proportion of non-adherence to ART among HIV-positive MSM were 28.6% and 10.5% with and without methamphetamine use, respectively. After controlling for demographics, illicit drug use, and co-morbidities, methamphetamine use during the prior three months was associated with a higher risk of non-adherence to ART (adjusted odds ratio = 3.08; 95% confidence intervals: 1.24–7.69). Compared with HIV-positive MSM with non-adherence to ART, HIV-positive MSM with good adherence to ART had a higher CD4 counts and were more likely to achieve an undetectable viral load. Since poor adherence to ART is associated with an increased HIV viral load and the risk of HIV transmission to others, our study suggests that it is imperative to screen HIV-positive patients for methamphetamine use and to provide effective therapy to reduce methamphetamine use and the associated non-adherence to ART.

## Introduction

As of 2018, there are 37.9 million people living with HIV/AIDS (PLWHA) worldwide^1^. The widespread use of antiretroviral therapy (ART) has significantly decreased the risk of opportunistic infections and mortality among PLWHA^2,3^. Although ART could markedly improve the survival in PLWHA, treatment compliance to ART plays an important role in determining the treatment success in this population^4,5^. Poor adherence to ART not only increases the risk of mortality^6^, but also causes HIV transmission to others^7^.

In 2014, the Joint United Nations Programme on HIV/AIDS and the World Health Organization announced that HIV pandemics must be stopped by 2030. To reach this goal, 95% of PLWHA should have access to HIV treatment and 95% of PLWHA accessing ART must achieve undetectable levels of viral load by 2030^8^. A previous study showed that PLWHA achieving and maintaining virological success requires an adherence rate of approximately 95%^9^.

In Taiwan, the first HIV infection was detected in 1984 among men who have sex with men (MSM)^10^. Since then, the number of PLWHA in Taiwan has increased gradually. At the end of 2018, the number of PLWHA has reached 37,602, of which 63.7% were MSM^10^. Although free-of-charge ART has been offered for all HIV-positive individuals since 1997^11^, it is estimated that around 80% of all HIV-positive patients in Taiwan received ART^10^. Moreover, among the treated patients, only approximately 80% achieved undetectable levels of viral load levels^10^.

Active illicit drug use is highly prevalent among PLWHA and has been associated with decreased access to HIV treatment and increased mortality^12^. Methamphetamine is one of the major illicit drugs used in PLWHA, particularly among MSM^13,14^. A previous study showed that methamphetamine use in HIV-positive MSM led to increased risky sexual behavior and caused the psychosocial impairment and memory loss^15^, which may decrease adherence to ART. However, studies to determine the association between methamphetamine use and adherence to ART are limited and report inconsistent findings^14,16,17^.

Non-adherence to ART is a major barrier to achieving the suppression of the HIV viral load. Identifying high-risk groups who do not display good adherence to ART could provide important information to improve HIV continuum care programs in Taiwan. This study therefore investigated the impact of methamphetamine use on ART adherence among HIV-positive MSM in Taiwan.

## Methods

### Study population and eligibility

This study consecutively recruited HIV-positive patients from Taipei City Hospital (TCH) HIV clinics between December 2018 and October 2019. TCH HIV clinics represent the largest HIV care center in Taiwan and provides ART for all Taiwanese HIV-positive individuals. Our study enrolled study participants who were 18 years of age or older, were receiving ART, and had provided written informed consent. If a study participant agreed, the case manager interviewed the participant about their treatment adherence using the Medication Adherence Report Scale (MARS-5)^18^. Study participants who completed the survey were compensated with a coupon of US$ 3 for their time.

To determine the factors associated with non-adherence in HIV-positive MSM, this study included PLWHA who were men who had sex with men and who had been receiving ART. Since the average time to first virologic suppression after initiation of ART was two months^19^, this study only included HIV-positive MSM who had been receiving ART for more than two months in the analysis. This study was approved by the Institutional Review Board of TCH (no. TCHIRB-10612120) and all interview with the study participants were performed in accordance with TCH IRB guidelines and regulations.

### Assessments of treatment adherence

Treatment adherence in PLWHA was assessed using the MARS-5^18,20–24^. The MARS has been used as a self-reported measure of adherence for a number of chronic diseases (e.g., heart failure^21^, asthma^23^, and constipation^20^) and has been proven to have good reliability and validity^18^. The MARS-5 consists of five common patterns of non-adherent behavior that respondents were asked to rate on a 5-point Likert scale (1 = always, 2 = often, 3 = sometimes, 4 = rarely, and 5 = never) (Table S1). The first statement of the MARS-5 addresses unintentional non-adherence (“I forget to take my anti-retroviral drugs”), whereas the other four statements question intentional non-adherence^20^. Scores were summed and, totals ranged from 5 to 25, with lower scores indicating lower self-reported adherence. There is no consensus on which cut-off value to use for dichotomizing the MARS-5 score; cut-off values in the literature have ranged from 20 to 25^20–26^. We chose to define non-adherence to ART as a MARS-5 score of <23, a cut-off value that commonly has been used for dichotomization of the MARS-5 in previous studies^20–24^.

### Data collection

At the time of study enrollment, consenting participants completed a face-to-face interview administered by the trained case manager using a standardized questionnaire. The average duration of the interview was approximately 40 minutes. Participants who completed the quantitative adherence survey were represented by a unique study ID number, not the patient's name or other personally identifying information. The questionnaire used in the interview survey collected information on participants’ MARS-5 score, socio-demographics, illicit drug use, comorbidities (e.g., depressive disorder), and history of sexually-transmitted diseases. Socio-demographic characteristics included age, sexual preference, income, and education. Sexual preference included heterosexual, homosexual, and bisexual. Illicit drug use information included questions on the use of methamphetamine, ecstasy, gamma hydroxybutyrate (GHB), amyl nitrite, marijuana, morphine, and cocaine during the prior three months. Other information concerning substance use (e.g., benzodiazepines) was also collected during the face-to-face interview. Participants’ history of sexually-transmitted diseases included questions on syphilis, gonorrhea, and genital warts. Viral load and CD4 counts in study participants were measured within 3 months of enrollment.

### Outcome variable

The primary outcome of this study was participants’ adherence to ART, which was determined by the MARS-5 score. Non-adherence and good adherence to ART were defined as a MARS-5 score of <23 and ≥23 respectively^20–24^.

### Main explanatory variable

The main explanatory variable was participants’ methamphetamine use during the prior three months. Methamphetamine use was investigated by asking participants: “Have you used methamphetamine during the prior three months?”

### Statistical analysis

First, the demographic data of the study participants were analyzed. Continuous data are presented as the mean (standard deviation [SD]), and the two-sample *t*-test was used for comparisons between groups. Categorical data were analyzed using the Pearson χ^2^ test, where appropriate.

Logistic regression was used to assess the univariate and multivariate associations of selected factors with adherence to ART. All variables found to be significant (*P* < 0.10) through univariate analysis were considered for inclusion in multivariate analysis. Forward stepwise regression was performed to produce the final model, which included the factors with *P* < 0.05. Odds ratios (OR) and adjusted odds ratios (AOR) with 95% confidence intervals (CI) were reported to show the strength and direction of these associations.

We conducted a sensitivity analysis to evaluate the association between any illicit drug use and non-adherence to ART in MSM living with HIV. All data management and analyses in this study were performed using the SAS 9.4 (SAS Institute, Cary, NC) and SPSS 19.0 (SPSS, Chicago IL, USA) software packages.

## Results

### Participant selection

This study consecutively recruited 368 HIV-positive MSM who were evaluated for the adherence to ART between December 2018 and October 2019. After excluding those who had received ART for less than two months (*n* = 17), the remaining 351 HIV-positive MSM were included in the analysis. The overall mean (SD) age was 37.1 (8.4) years; mean (SD) duration of receiving ART at the time of enrollment was 89.7 (68.1) months; 16.0% of the participants had used methamphetamine in the prior three months; and 13.4% reported non-adherence to ART.

### Characteristics of HIV-positive MSM with and without methamphetamine use

Table 1 shows the characteristics of HIV-positive MSM with and without methamphetamine use during the prior three months. Compared with HIV-positive MSM without methamphetamine use, those who used methamphetamine reported higher level of non-adherence to ART (28.6% versus 10.5%). Moreover, HIV-positive MSM with methamphetamine use were younger, had lower income, and were also more likely to have used ecstasy and GHB in the prior three months. Concerning comorbidities, HIV-positive MSM with methamphetamine use were more likely to have depressive disorder and to have been infected with syphilis or gonorrhea.

**Table 1:** Characteristics of the HIV-positive MSM, by treatment adherence.

Characteristics	No. (%) of participants^*^		P value
	HIV-positive MSM without methamphetamine use^a^, n=295	HIV-positive MSM with methamphetamine use^a^, n=56	
**Socio-demographics**			
Age, yr			
Mean (SD)	37.5 (8.5)	34.8 (7.5)	0.026
15–39	186 (63.1)	44 (78.6)	0.025
≥40	109 (36.9)	12 (21.4)	
Education level completed			
≤High school	81 (27.5)	13 (23.2)	0.511
University or above	214 (72.5)	43 (76.8)	
Income level			
Low	29 (9.8)	13 (23.2)	0.002
Intermediate	132 (44.7)	29 (51.8)	
High	134 (45.5)	14 (25.0)	
**Illicit drug use**			
Ecstasy^a^			
No	285 (96.6)	48 (85.7)	0.001
Yes	10 (3.4)	8 (14.3)	
GHB^a^			
No	294 (99.7)	39 (69.6)	<0.001
Yes	1 (0.3)	17 (30.4)	
Any alcohol use			
No	156 (52.9)	29 (51.8)	0.88
Yes	139 (47.1)	27 (48.2)	
Smoking			
No	176 (59.7)	36 (64.3)	0.517
Yes	119 (40.3)	20 (35.7)	
**Comorbidities**			
Depressive disorder			
No	260 (88.1)	40 (71.4)	0.001
Yes	35 (11.9)	16 (28.6)	
History of syphilis			
No	141 (47.8)	15 (26.8)	0.004
Yes	154 (52.2)	41 (73.2)	
History of gonorrhea infection			
No	268 (88.8)	44 (78.6)	0.036
Yes	33 (11.2)	12 (21.4)	
History of warts			
No	245 (83.1)	45 (80.4)	0.626
Yes	50 (16.9)	11 (19.6)	
CD4 count, cells/mm^3^			
<200	8 (2.7)	0	0.186
200–499	111 (37.6)	27 (48.2)	
≥500	176 (59.7)	29 (51.8)	
HIV-1 RNA, copies/ml			
HIV-1 RNA<40	271 (91.9)	48 (85.7)	0.143
HIV-1 RNA≥40	24 (8.1)	8 (14.3)	
Treatment adherence to ART			
Non-adherence	31 (10.5)	16 (28.6)	<0.001
High adherence	264 (89.5)	40 (71.4)	

^*^Unless stated otherwise. ^a^During the prior 3 months. MSM, men who have sex with men; SD, standard deviation; GHB, Gamma Hydroxybutyrate; ART, highly active anti-retroviral therapy.

### Univariate analysis for the factors associated with non-adherence to antiretroviral therapy

Table 2 shows the results of the univariate analysis for the factors associated with non-adherence to ART in MSM living with HIV. The variables significantly associated with non-adherence to ART in the univariate analysis included methamphetamine use during the prior three months, income level, depressive disorder, CD4 count, and plasma viral load.

**Table 2:** Univariate analyses of factors associated with non-adherence to antiretroviral treatment among HIV-infected MSM.

Characteristic	Number of patients	Non-adherence to ART	Univariate analysis
		n (%)	OR (95% CI)
Methamphetamine use in prior 3 months			
No	295	31 (10.5)	1
Yes	56	16 (28.6)	3.41 (1.71–6.78)^***^
**Demographics**			
Age, yr			
15–39	230	33 (14.3)	1
≥40	121	14 (11.6)	0.78 (0.40–1.52)
Education level completed			
≤High school	94	15 (16.0)	1
University or above	257	32 (12.5)	0.75 (0.39–1.46)
Income level			
Low	42	9 (21.4)	1
Intermediate	161	25 (15.5)	0.67 (0.29–1.58)
High	148	13 (8.8)	0.35 (0.14–0.90)^*^
**Illicit drug use**			
Ecstasy^a^			
No	333	45 (13.5)	1
Yes	18	2 (11.1)	0.80 (0.18–3.60)
GHB^a^			
No	333	43 (12.9)	1
Yes	18	4 (22.2)	1.93 (0.61–6.13)
Any alcohol use			
No	185	24 (13.0)	1
Yes	166	23 (13.9)	1.08 (0.58–2.00)
Smoking			
No	212	22 (10.4)	1
Yes	139	25 (18.0)	1.89 (1.02–3.51)
**Comorbidities**			
Depressive disorder			
No	300	33 (11.0)	1
Yes	51	14 (27.5)	3.06 (1.50–6.25)^**^
History of syphilis			
No	156	19 (12.2)	1
Yes	195	28 (14.4)	1.21 (0.65–2.26)
History of gonorrhea infection			
No	306	38 (12.4)	1
Yes	45	9 (20.0)	1.76 (0.79–3.95)
History of warts			
No	290	37 (12.8)	1
Yes	61	10 (16.4)	1.34 (0.63–2.87)
CD4 count, cells/mm^3^			
<200	8	3 (37.5)	1
200–499	138	24 (17.4)	0.35 (0.08–1.57)
≥500	205	20 (9.8)	0.18 (0.04–0.81)^*^
HIV-1 RNA, copies/ml			
HIV-1 RNA<40	319	33 (10.3)	1
HIV-1 RNA≥40	32	14 (43.8)	6.74 (3.07–14.79)^***^

^*^<0.05; ^**^<0.01; ^***^<0.001. MSM, men who have sex with men; ART, antiretroviral treatment; AOR: adjusted odds ratio; CI: confident interval; GHB, Gamma Hydroxybutyrate.

### Multivariate analysis for the factors associated with non-adherence to antiretroviral therapy

Factors associated with non-adherence to ART in the univariate analysis (*p* < 0.10) were considered for inclusion in the multivariate analysis. After controlling for demographics, illicit drug use, and co-morbidities, methamphetamine use during the prior three months was associated with a higher risk of non-adherence to ART (AOR = 2.80; 95% CI: 1.31–5.97; *p* = 0.008; Table 3). Other independent risk factor for non-adherence to ART was depressive disorder. Compared with HIV-positive MSM with undetectable viral load, those with detectable viral load had a lower adherence to ART. Moreover, HIV-positive MSM with a CD4 count ≥500 had a higher adherence to ART than those with a CD4 count <200. Furthermore, a positive trend was noted between increasing CD4 counts and higher adherence to ART (*p* = 0.018).

**Table 3:** Multivariate analyses of factors associated with non-adherence to antiretroviral treatment among MSM living with HIV.

Characteristic	AOR (95% CI)
Methamphetamine use in prior 3 months	2.80 (1.31–5.97)^**^
Depressive disorder	3.37 (1.53–7.44)^**^
CD4 count, cells/mm^3^	
<200	1
200–499	0.24 (0.05–1.26)
≥500	0.15 (0.03–0.80)^*^
HIV-1 RNA, copies/ml	
HIV-1 RNA<40	1
HIV-1 RNA≥40	6.85 (2.93–16.01)^***^

^*^<0.05; ^**^<0.01; ^***^<0.001. MSM, men who have sex with men; AOR: adjusted odds ratio; CI: confident interval.

### Sensitivity analysis for the association between illicit drug use and non-adherence to antiretroviral therapy

This study evaluated the association between any illicit drug use and non-adherence to ART in MSM living with HIV. After controlling for demographics and co-morbidities, MSM living with HIV who used any of illicit drugs were more likely to have non-adherence to ART compared to those who did not use any illicit drugs (AOR = 2.65; 95% CI: 1.27–5.53; *p* = 0.009; Supplementary Table 2).

## Discussion

This study interviewed 351 HIV-positive MSM and found that methamphetamine use during the prior three months was associated with a higher risk of non-adherence to ART. Moreover, compared with HIV-positive MSM with non-adherence to ART, HIV-positive MSM with adherence to ART were more likely to have a higher CD4 counts and were more likely to achieve undetectable viral load.

Methamphetamine use could cause the psychosocial impairment^15^, which may decrease adherence to ART. However, studies evaluating the association between methamphetamine use and adherence to ART in PLWHA are limited and had inconsistent findings^14,16,17^. A study in Canada found that methamphetamine injection was not significantly associated with adherence to ART in injecting drug users living with HIV^17^. However, a cross-sectional study in the United States showed that methamphetamine use was associated with a significant reduction in ART adherence among HIV-positive gay and bisexual men^16^. Another study involving 150 HIV-positive individuals found that PLWHA with a combination of methamphetamine and cocaine use had significantly poorer adherence to ART than those with cocaine use only^14^. Our study found that, after adjusting for demographics, illicit drug use, and co-morbidities, methamphetamine use during the prior three months was an independent risk factor for non-adherence to ART. As poor adherence to ART is associated with an increased HIV viral load^27^, treatment resistance^4^, mortality^6^, and the risk of HIV transmission to others^7^, future HIV care continuum programs should focus on PLWHA who use methamphetamine to improve treatment adherence and health outcomes.

Methamphetamine-induced neuronal injury and neurocognitive deficits^28–30^ may cause the non-adherence to ART in HIV-positive MSM. A previous perfusion magnetic resonance imaging study showed that methamphetamine use caused decreased cerebral blood flow and neuronal injury, which resulted in memory and cognitive deficits^28^. Another positron emission tomography study found that methamphetamine use caused dopamine transporter reduction that lead to the neurocognitive impairment^29^. Furthermore, a recent meta-analysis showed that individuals with methamphetamine use disorder had moderate deficits in brain cognition^30^. Neurocognitive impairment has been negatively associated with treatment adherence in PLWHA^31,32^. The findings of our study suggest that PLWHA who use methamphetamine should be targeted to improve adherence to antiretroviral therapy.

Methamphetamine is the prevalent recreational drug associated with “chemsex” in MSM^13^. A prior report in Taiwan showed that the proportions of methamphetamine use were 22.3% and 11.7% in HIV-positive and HIV-negative MSM, respectively^33^. Our study found that 16.0% of HIV-positive MSM had used methamphetamine in the prior three months, which was higher than the 7.8% of HIV-positive MSM in the United Kingdom^34^. In Taiwan, there was a surge in methamphetamine use over the last decade^35^. To treat individuals with methamphetamine use disorder, Taiwan government implemented a mandatory addiction treatment modality that includes group psychotherapy, bi-weekly counseling, and monitoring of urinalysis for methamphetamine use^36^. A previous report showed that the mandatory treatment modality could significantly reduce methamphetamine use in individuals with methamphetamine use disorder^36^. Since methamphetamine use in MSM could cause poor adherence to ART and increase the risk of HIV transmission to others, the findings of our study suggest that it is imperative to provide effective therapies^36^,^37^ for MSM who abuse methamphetamine to improve their adherence to ART.

This study has several limitations. First, the cross-sectional study design did not allow us to determine the causality between methamphetamine use and adherence to ART. Future longitudinal studies are needed to investigate the impact of methamphetamine use on ART adherence. Second, methamphetamine use in PLWHA was self-reported and may have been under-reported owing to social desirability bias. However, this would be less likely because of the anonymous questionnaire survey. Third, a limited number of participants used amyl nitrite, marijuana, morphine, cocaine, and benzodiazepines in this study, which precluded this analysis from determining the association between ART adherence and the use of these substances. Finally, the external validity of our findings may be a concern because almost all our participants were Taiwanese. Therefore, the generalizability of our results to other non-Asian ethnic groups requires further verification. Nevertheless, our findings suggest new avenues for future research.

In summary, this study found that 13.4% of HIV-positive MSM were non-adherent to ART. After adjusting for demographics, illicit drug use, and co-morbidities, methamphetamine use during the prior three months was an independent risk factor for non-adherence to ART. As methamphetamine is a prevalent recreational drug among HIV-positive MSM, it is imperative to screen for methamphetamine use and provide effective therapy to reduce methamphetamine use and the associated non-adherence to ART.

## Supplementary information

Supplementary Table S1.

Supplementary Table 2.
