# Supplementary material for: Methamphetamine Use Associated with Non-adherence to Antiretroviral Treatment in Men Who Have Sex with Men
Source: Sci Rep. 2020 Apr 28;10:7131. doi: 10.1038/s41598-020-64069-2 (PMC7188802; doi:10.1038/s41598-020-64069-2)
Supplement: Supplementary file 1 — Supplementary Table S1. [file 41598_2020_64069_MOESM1_ESM.docx]

| Table S1: Medication Adherence Report Scale (MARS-5). | | |
| --- | --- | --- |
| Item number | Question | Response |
| MARS-Q1 | I forget to take my anti-retroviral drugs | □ (1) Always  □ (2) Often  □ (3) Sometimes □ (4) Rarely  □ (5) Never |
| MARS-Q2 | I alter the dose of my anti-retroviral drugs | □ (1) Always  □ (2) Often  □ (3) Sometimes □ (4) Rarely  □ (5) Never |
| MARS-Q3 | I stop taking my anti-retroviral drugs for a while | □ (1) Always  □ (2) Often  □ (3) Sometimes □ (4) Rarely  □ (5) Never |
| MARS-Q4 | I decide to skip a dose of my anti-retroviral drugs | □ (1) Always  □ (2) Often  □ (3) Sometimes □ (4) Rarely  □ (5) Never |
| MARS-Q5 | I take less anti-retroviral drugs than instructed | □ (1) Always  □ (2) Often  □ (3) Sometimes □ (4) Rarely  □ (5) Never |
