# Supplementary material for: Methamphetamine Use Associated with Non-adherence to Antiretroviral Treatment in Men Who Have Sex with Men
Source: Sci Rep. 2020 Apr 28;10:7131. doi: 10.1038/s41598-020-64069-2 (PMC7188802; doi:10.1038/s41598-020-64069-2)
Supplement: Supplementary file 2 — Supplementary Table 2. [file 41598_2020_64069_MOESM2_ESM.docx]

| **Characteristic** | **Number of patients** | **Non-adherence to ART** | **Univariate analysis** | **Multivariate analysis^a^** |
| --- | --- | --- | --- | --- |
|  |  | **n (%)** | **OR (95% CI)** | **AOR (95% CI)** |
| Any illicit drug use^b^ |  |  |  |  |
| No | 285 | 30 (10.5) | 1 | 1 |
| Yes | 66 | 17 (25.8) | 2.95 (1.51-5.76)^**^ | 2.65 (1.27-5.53)** |
| Depressive disorder |  |  |  |  |
| No | 300 | 33 (11.0) | 1 | 1 |
| Yes | 51 | 14 (27.5) | 3.06 (1.50-6.25)^**^ | 3.46 (1.58-7.62)** |
| CD4 count, cells/mm^3^ |  |  |  |  |
| <200 | 8 | 3 (37.5) | 1 | 1 |
| 200-499 | 138 | 24 (17.4) | 0.35 (0.08-1.57) | 0.24 (0.04-1.25) |
| ≥500 | 205 | 20 (9.8) | 0.18 (0.04-0.81)^*^ | 0.15 (0.03-0.78)* |
| HIV-1 RNA, copies/ml |  |  |  |  |
| HIV-1 RNA<40 | 319 | 33 (10.3) | 1 | 1 |
| HIV-1 RNA≥40 | 32 | 14 (43.8) | 6.74 (3.07-14.79)^***^ | 7.11 (3.04-16.61)*** |
| Supplementary table 2 Multivariate analyses of the association between any illicit drug use and non-adherence to antiretroviral treatment among MSM living with HIV. ^*^<.05; ^**^<.01; ^***^<.001. MSM, men who have sex with men; ART, antiretroviral treatment; AOR: adjusted odds ratio; CI: confident interval; ^a^adjusting for demographics and co-morbidities; ^b^including methamphetamine, ecstasy, and gamma hydroxybutyrate (GHB). | | | | |
